# Hyperuricemia is associated with cardiovascular diseases clustering among very elderly women - a community based study in Chengdu, China

**DOI:** 10.1038/s41598-017-01042-6

**Published:** 2017-04-20

**Authors:** Gang Huang, Jun-bo Xu, Ting-jie Zhang, Xiao-li Nie, Qiu Li, Ya liu, Yan Lv, Rui-lian Wen, Lei Yang, Bao-yu Zhao

**Affiliations:** 1 Cardiovascular and Metabolic Disease Center, The Second People’s Hospital of Chengdu, Chengdu, China; 2 Department of Cardiology, The Second People’s Hospital of Chengdu, Chengdu, China

## Abstract

Cardiovascular epidemiological features among very elderly Chinese are still uncertain. This study aimed to describe the distribution of cardiovascular diseases and sex difference, and investigate potential risk factors for diseases clustering among very elderly Chinese. From May 2013 to May 2015, a total of 1056 very elderly were sampled in this cross-sectional study. Demographic characteristics collection, physical examination and biochemical analysis were performed. Totally, 1038 participants (men: 49.8%) with a median age of 83.0 years (age range: 80.0–100.0 years) were included. In this very elderly group, the prevalences of hypertension, diabetes mellitus, renal dysfunction, hyperuricemia, obesity, visceral obesity, and hypercholesterolaemia were 76.9%, 20.0%, 23.3%, 33.0%, 9.4%, 54.5% and 35.8%, respectively. About 17.5% of very elderly (men vs. women: 15.1% vs 19.8%, *p* = 0.007) have ≥3 cardiovascular diseases clustering. Logistic analysis found that hyperuricemia (odds ratio 3.850, 95%CI 2.189–6.770) was associated with of cardiovascular diseases clustering among very elderly women. Prevalences of prehypertension, hyperuricemia, visceral obesity and dyslipidaemia are apparent in very elderly women, while diabetes mellitus, renal dysfunction are common in very elderly men. Women are more likely to have ≥3 cardiovascular diseases. Hyperuricemia is associated with cardiovascular diseases clustering among very elderly women.

## Introduction

Cardiovascular diseases are predominated causes of death in the world^1^. When there were no improvement on current trends in smoking, hypertension, diabetes mellitus (DM) and obesity, premature cardiovascular deaths would increase about 32% in 2025, and 60% of these deaths would occur in South Asia, East Asia, and Southeast. Moreover, there would be no detectable change in premature cardiovascular diseases mortality by 2025 in China^2^.

In China, cardiovascular disease is not only the first burden of disease^3^, but also accounts for about 38% total death in all ages^4^. Stroke, ischemic heart disease, hypertensive heart disease, and DM are all among the top ten causes of death for Chinese^3^.

As China is becoming a aging society rapidly, there is a growing urgency to tackle cardiovascular and metabolic diseases through effective prevention and treatment strategies, informed by precise estimates of disease prevalence and burden. Most of these current epidemic data are from population younger than 80 years^5–7^, although in 2010 the proportion of elderly Chinese is already 1.57%^8^. There is relatively few study on cardiovascular disease risk factors in very elderly Chinese. Hence, the aim of this study was to describe the distribution of cardiovascular diseases and sex difference, and investigate potential risk factors for diseases clustering among very elderly Chinese in Chengdu.

## Results

### Baseline characteristics

Totally, 1056 participants were enrolled in this survey. The overall response rate was 92.6%. Finally, 18 participants with incomplete data were excluded and therefore 1038 participants (men vs women: 49.8% vs 50.2%) were included in final analysis. The median age was 83.0 years (lower quartile: 81.0, upper quartile: 85.0, age range: 80.0–100.0 years), in which men were slightly older than women (*p* = 0.342). Among all participants, more than 10% were current smokers and 8% of them were current alcohol drinker. Not surprisingly, both proportions in men were much significantly higher than in women (both *p* < 0.001). Nearly 70% of these men were educated in a primary school or a middle/high school. While, more than 30% of women were illiterate compared with 11% in men (*p* < 0.001). Moreover, the proportion of women educated in middle/high school and college/university were significantly lower than which in men (both *p* < 0.001) (Table 1).

**Table 1:** Demographic characteristics and cardiovascular risk factors.

	All	Men (N = 517)	Women (N = 521)	p value
Age, years	83.6 ± 3.4	83.6 ± 3.3	83.5 ± 3.4	0.191
	83.0 (81.0, 85.0)	83.0 (81.0, 85.0)	83.0 (81.0, 85.0)	
Han Ethnicity n, (%)	1029(99.1)*	513(99.2)	516(99.0)	1.000
Current Smoker n, (%)	116(11.2)*	93(18.0)	23(4.4)	<0.001
Current Drinking n, (%)	87(8.4)*	78(15.1)	9(1.7)	<0.001
Education				
Illiterate n, (%)	249(24.0)*	60(11.6)	189(36.3)	<0.001
Primary School n, (%)	312(30.0)*	154(29.8)	158(30.3)	0.913
Middle/High School n, (%)	336(32.4)*	210(40.6)	126(24.1)	<0.001
College/University n, (%)	141(13.6)*	93(18.0)	48(9.3)	<0.001
Blood pressure				
SBP mmHg	146.4 ± 20.6	145.0 ± 20.0	148.0 ± 21.0	0.038
DBP mmHg	74.1 ± 11.9	75.0 ± 11.6	73.1 ± 12.1	0.006
PP mmHg	72.5 ± 17.1	70.2 ± 16.8	75.2 ± 17.1	<0.001
Heart rate, bpm	70.0 ± 9.0	68.4 ± 9.5	71.3 ± 8.3	0.003
Height, cm	154.9 ± 10.1	161.9 ± 7.3	147.9 ± 7.2	<0.001
Body weight, kg	55.7 ± 10.9	60.4 ± 10.0	50.9 ± 9.6	<0.001
BMI, kg/m^2^	23.1 ± 3.7	23.0 ± 3.5	23.2 ± 4.0	0.484
WC, cm	87.5 ± 10.6	87.7 ± 10.2	87.4 ± 10.9	0.684
WHtR	0.57 ± 0.08	0.54 ± 0.07	0.59 ± 0.08	<0.001
FG, mmol/L	5.53 ± 1.35	5.65 ± 1.45	5.40 ± 1.22	0.004
Lipids				
TC, mmol/L	4.87 ± 0.99	4.67 ± 0.94	5.09 ± 1.00	<0.001
TG, mmol/L	1.34 ± 0.57	1.30 ± 0.57	1.38 ± 0.56	0.005
LDL–C, mmol/L	2.58 ± 0.74	2.49 ± 0.72	2.69 ± 0.75	<0.001
HDL–C, mmol/L	1.60 ± 0.44	1.52 ± 0.41	1.69 ± 0.46	<0.001
UA, μmol/L	350.1 ± 84.5	366.9 ± 81.9	331.2 ± 85.6	<0.001
Creatinine, μmol/L	104.1 ± 32.4	111.3 ± 34.2	95.8 ± 28.0	<0.001
e GFR, ml/(min · 1.73 m^2^)	58.7 ± 13.9	62.1 ± 14.0	56.6 ± 12.7	<0.001

BMI, body mass index; DBP, diastolic blood pressure; eGFR, estimated goblet filtration rate; FG, fasting glucose; HDL–C, high–density lipoprotein cholesterol; LDL-C, low–density lipoprotein cholesterol; PP, pulse pressure; SBP, systolic blood pressure; TC, total cholesterol; TG, triglycerides; UA, Uric acid; WC, waist circumference; WHtR, waist–to-height ratio. Data are presented as mean ± standard deviation for continuous variables and as frequencies (percentages) for categorical variables, age also presented as median (interquartile). eGFR was calculated according to Cockcroft-Gault equation. *Prevalences are standardized for age and sex.

### Mean levels of cardiovascular risk factors

Mean levels of systolic blood pressure (SBP), diastolic blood pressure (DBP), and pulse pressure (PP) in all participants were 146.4 ± 20.6 mmHg, 74.1 ± 11.9 mmHg, and 72.5 ± 17.1 mmHg, separately. In women, there were relatively higher mean levels of SBP (*p* = 0.038), PP (*p* < 0.001), and heart rate (*p* = 0.003), while a lower mean DBP level (*p* = 0.006), respectively.

Compared with women, very elderly men were higher (161.9 ± 7.3 vs. 147.9 ± 7.2 cm, *p* < 0.001), and heavier (60.4 ± 10.0 vs. 50.9 ± 9.6 Kg, *p* < 0.001). However, there were no significant difference in body mass index (BMI) and waist circumference (WC) between very elderly men and women (both *p* > *0*.*05*). While, the mean waist–to-height ratio was significant lower in very elderly men (0.54 ± 0.07 vs. 0.59 ± 0.08, *p* < *0*.*001*).

The overall mean fast glucose (FG) of all participants was 5.53 ± 1.35 mmol/L (men vs women: 5.65 ± 1.45 vs 5.40 ± 1.22 mmol/L, *p* = 0.004). Mean levels of total cholesterol (TC), triglycerides (TG), low-density lipoprotein cholesterol (LDL-C), and high-density lipoprotein cholesterol (HDL-C) in all participants were 4.87 ± 0.99 mmol/L, 1.34 ± 0.57 mmol/L, 2.58 ± 0.74 mmol/L, and 1.60 ± 0.44 mmol/L, respectively. Furthermore, all mean levels of these parameters were significantly higher in very elderly women than in men (all *p* < 0.05).

And mean levels of urine acid (UA) and Creatinine in all very elderly participants were 350.1 ± 84.5 μmol/L and 104.1 ± 32.4 μmol/L. Compared with very elderly women, men had a significantly higher UA (366.9 ± 81.9 vs 331.2 ± 85.6 μmol/L, *p* < 0.001) and Creatinine levels (111.3 ± 34.2 vs 95.8 ± 28.0 μmol/L, *p* < 0.001) (Table 1).

### Estimated prevalence of cardiovascular abnormalities

Estimated prevalences of pre-hypertension and hypertension among very elderly participants were 16.6% and 76.9%, respectively. While, both prevalences were not significantly different between sexes, although both of them were a bit higher in very elderly women (both *p* > 0.05). The estimated prevalence of DM was about 20.0% among overall very elderly participants. The prevalence of DM among very elderly men did not differ significantly from women (21.4% vs 18.8%, *p* = 0.285). The estimated prevalence of renal dysfunction among the very elderly participants was 23.3%, which was doubled in men than in women (32.6% vs 14.1%, *p* < 0.001). As well, we could not ignore that the prevalence of hyperuricemia among very elderly was as high as 33.0%, which was even higher in women than that in men (35.6% vs 30.2%, *p* > 0.05). We also presented the estimated prevalence of renal dysfunction (eGFR < 60 ml/(min · 1.73 m^2^)) according to Cockcroft-Gault equation in Table 2.

**Table 2:** Estimated prevalence of cardiovascular abnormalities.

	All *n, % (95%CI)	Men (N = 517) n, % (95%CI)	Women (N = 521) n, % (95%CI)	p value
Pre Hypertension	173, 16.6(14.4–18.9)	83, 16.0(12.9–19.2)	90, 17.2(14.0–20.5)	0.598
Hypertension	797, 76.9(74.2–79.4)	391, 75.6(71.9–79.3)	406, 78.0(74.4–81.5)	0.353
Self-reported	545, 68.4(65.2–71.6)	261, 66.8(62.1–71.4)	284, 70.0(65.5–74.4)	0.332
IFG	61, 5.8(4.4–7.3)	37, 7.1(4.9–9.4)	24, 4.6(2.8–6.4)	0.130
DM	209, 20.0(17.6–22.5)	111, 21.4(17.9–25.0)	98, 18.8(15.5–22.2)	0.285
Self-reported	123, 58.9(52.2–65.5)	58, 52.3(43.0–61.5)	65, 66.3(57.0–75.7)	0.039
Renal dysfunction	242, 23.3(20.7–25.9)	169, 32.6(28.6–36.6)	73, 14.1(11.1–17.1)	<0.001
**	574, 55.3(52.3–58.3)	220, 42.6(38.3–46.8)	354, 67.9(63.9–72.0)	<0.001
Hyperuricemia	342, 33.0(30.1–35.8)	156, 30.2(26.2–34.1)	185, 35.6(31.4–39.6)	0.075
Persistant AF	54, 5.2(3.8–6.5)	31, 5.9(3.9–7.9)	23, 4.5(2.7–6.3)	0.316
Persistant Af	3, 0.3(0.0–0.6)	1, 0.2(−0.2–0.6)	2, 0.4(−0.1–0.9)	0.604
I AVB	64, 6.3(4.8–7.8)	38, 7.4(5.1–9.7)	27, 5.1(3.2–7.0)	0.137
II/III AVB	2, 0.2(−0.1–0.4)	2, 0.4(−0.1–0.9)	0	0.345
RBBB	106, 10.2(8.3–12.0)	55, 10.6(7.9–13.3)	51, 9.8(7.2–12.4)	0.663
LBBB	6, 0.6(0.1–1.0)	4, 0.8(0.0–1.5)	2, 0.4(−0.1–0.9)	0.788
Overweight	312, 30.0(27.3–32.8)	156, 30.2(26.2–34.1)	156, 29.9(26.0–33.9)	0.920
Obesity	98, 9.4(7.6–11.1)	41, 7.9(40.8–49.4)	57, 10.9(8.1–13.4)	0.138
Visceral Obesity	560, 54.5(51.5–57.6)	233, 45.1(5.6–10.3)	327, 62.7(58.6–66.9)	<0.001
Hypercholesterolaemia	367, 35.8(32.9–38.8)	144, 27.9(24.0–31.7)	223, 42.8(38.6–47.1)	<0.001
Hypertrigleicemiea	222, 21.6(18.9–23.9)	97, 18.8(15.4–22.1)	125, 24.0(20.3–27.7)	0.048
Hyper-low density lipoproteinemia	152, 14.9(12.8–17.1)	59, 11.4(8.7–14.2)	93, 17.9(14.6–21.1)	0.004
Hypo-high density lipoproteinemia	69, 6.5(5.1–8.2)	46, 8.9(6.4–11.4)	23, 4.5(2.7–6.2)	0.008

AF, atrial fibrillation; Af, atrial flutter; AVB, atrial ventricular block; DM, diabetes mellitus; FG, fast glucose; IFG, impaired fasting glucose; LBBB, = left bundle brach block; RBBB, right bundle brach block. Data are presented as and as number, frequency (95% confidence interval). *Prevalences are standardized for age and sex. **Prevalence of renal dysfunction was defined as e GFR < 60 ml/(min · 1.73 m^2^) according to Cockcroft-Gault equation.

Compared with obesity, overweight and visceral obesity were more common in very elderly with a prevalence of 30.0% and 54.5%, respectively. Especially in very elderly women, the prevalence of visceral obesity was significantly higher than in men (62.7% vs 45.1%, *p* < 0.001).

As among the very elderly, estimated prevalences of persistent atrial fibrillation and three degrees atrial ventricular block were also higher in men than in women, while not significantly (all *p* > 0.05).

The estimated prevalence of hypercholesterolaemia in very elderly was one and half times as the prevalence of hypertrigleicemiea (35.8% vs 21.6%). And both prevalences were significantly higher in women than in men (both p < 0.05) (Table 2).

### Cluster of cardiovascular diseases

Among participants with DM, the prevalence of comorbid visceral Obesity was 63.2%, while among participants with hypertension, the prevalence of comorbid visceral Obesity was 57.3%, which was even lower (*p* = *0*.*129*). Among participants with both hypertension and DM, 54.1% of them had visceral Obesity and 27.3% of them had renal dysfunction. Prevalences of hypertrigleicemiea and hyperuricemia among participants with DM were higher than which among participants with hypertension, while the prevalence of hypercholesterolaemia was lower. Moreover, in participants with both hypertension and DM, prevalences of all other cardiovascular abnormalities were lower than in participants separately with hypertension or DM, except for renal function (Table 3).

**Table 3:** Percentage of comorbid abnormalities.

	Percentage n, % (95%CI)		
	Among Hypertension (N = 797)	Among DM (N = 209)	Among both hypertension and DM (N = 183)
Hypertension	—	173, 82.8(77.7–87.9)	—
DM	175, 22.0(19.1–24.8)	—	—
Renal dysfunction	194, 24.3(21.4–27.3)	55, 26.3(20.3–32.3)	50, 27.3(20.9–33.8)
Overweight	257, 32.2(29.0–35.5)	73, 34.9(28.5–41.4)	56, 30.6(23.9–37.3)
Obesity	87, 10.9(8.8–13.1)	32, 15.3(10.4–20.2)	19, 10.4(6.0–14.8)
Visceral Obesity	457, 57.3(53.9–60.8)	132, 63.2(56.6–69.7)	99, 54.1(46.9–61.3)
Hypercholesterolaemia	292, 36.6(33.3–40.0)	74, 35.4(28.9–41.9)	65, 35.5(28.6–42.5)
Hypertrigleicemiea	176, 22.1(19.2–25.0)	58, 27.8(21.7–33.8)	39, 21.3(15.4–27.2)
Hyperuricemia	285, 35.8(32.4–39.1)	80, 38.3(31.7–44.9)	62, 33.9(27.0–40.7)

DM, diabetes mellitus. Data are presented as frequencies (percentages) for categorical variables. All *p* value > 0.05, comparison between groups hypertension and DM.

Among all participants, percentages of one participant having 1, 2, or ≥3 of the 4 diseases, including hypertension, DM, renal dysfunction and dyslipidaemia (hypercholesterolaemia, hypertrigleicemiea, hyper-low density lipoproteinemia or hypo-high density lipoproteinemia) were 35.5%, 38.0%, and 17.5%, respectively. Furthermore, the percentage of one participant having ≥3 diseases in very elderly women was significantly higher than in men (women vs. men: 19.8% vs. 15.1%, p = 0.007).

Multiple logistic regression analysis found that hyperuricemia (odds ratio 3.850, 95%CI 2.189–6.770) is associated with clustering of ≥3 comorbid diseases (hypertension, DM, renal dysfunction, and dyslipidaemia) among very elderly women (Table 4).

**Table 4:** Logistic regression results of risk factors for ≥3 comorbid abnormalities.

	Total	Men	Women
Age (years)			
80–84	Ref.	Ref.	Ref.
85–89	0.743 (0.480–1.151)	0.872 (0.487–1.561)	0.569 (0.286–1.130)
≥90	0.604 (0.262–1.394)	0.715 (0.228–2.248)	0.443 (0.125–1.573)
Smoking			
No	Ref.	Ref.	Ref.
Yes	0.955 (0.498–1.830)	0.962 (0.461–2.008)	0.651 (0.136–3.115)
Drinking			
No	Ref.	Ref.	Ref.
Yes	0.812 (0.394–1.672)	0.653 (0.296–1.443)	0.761 (0.082–7.035)
Visceral Obesity			
No	Ref.	Ref.	Ref.
Yes	1.179 (0.804–1.730)	1.284 (0.767–2.150)	1.404 (0.747–2.639)
PP ≥ 60 mmHg			
No	Ref.	Ref.	Ref.
Yes	2.684 (1.464–4.920)*	3.961 (1.741–9.014)*	1.910 (0.763–4.777)
Hyperuricemia			
No	Ref.	Ref.	Ref.
Yes	2.066 (1.418–3.011)*	1.268 (0.730–2.205)	3.850 (2.189–6.770)*

Ref., reference category. Data are presented as odds ratio (95%CI). CI = confidence interval; Hyperuricemia defined as UA ≥ 416 μmol/L in men, UA ≥ 357 μmol/L in women. Visceral Obesity defined as waist circumference ≥90 cm in men, waist circumference ≥85 cm in women. Comorbid abnormalities included hypertension, diabetes mellitus, renal dysfunction, and dyslipidaemia. *P < 0.05 versus reference category.

**Figure 1:**
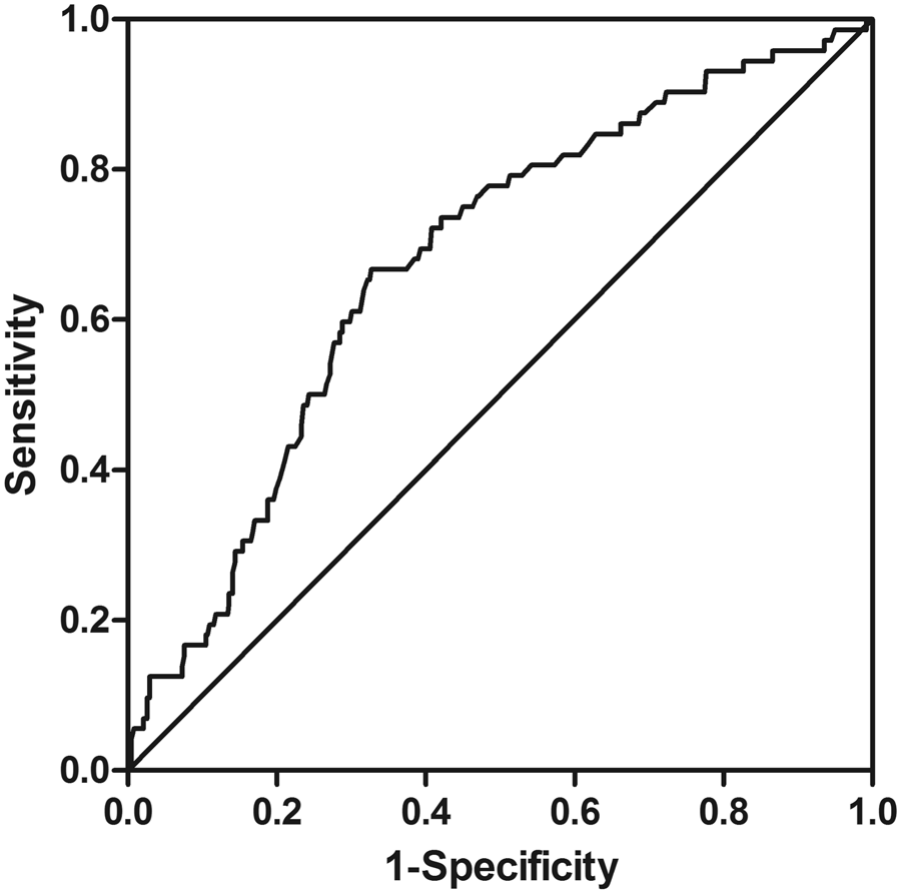
Receiver-operating characteristic curve (ROC) of UA predicting cardiovascular diseases clustering. ROC analysis yielded an optimal cutoff value of 352.5 μmol/L (p < 0.001) for very elderly women. The area under the ROC curve was 0.677 (95%CI 0.611–0.742).

Receiver-operating characteristic (ROC) analysis found that the optimal cutoff point for UA predicting cardiovascular diseases clustering was 352.5 μmol/L among very elderly women (p < 0.001) (Fig. 1). The sensitivity and specificity of UA ≥ 352.5 μmol/L for predicting AF were 66.7% and 67.3% among very elderly women.

## Discussion

To our knowledge, this survey is the first community population-based study providing general epidemiological information across wide spectrum of very elderly Chinese in Chengdu. This survey demonstrates as follows: (1) Cardiovascular risk factors are common among very elderly. (2) Sex differences of risk factors are apparent among very elderly, and women are more likely to cluster ≥3 diseases. (3) Hyperuricemia is associated with cardiovascular diseases clustering among very elderly women.

According to the newest report by World Health Organization (WHO), the life expectancy at birth for Chinese in 2015 is 76.1 years^9^. The average age of participants in this study is more than 83 years. Although much of the evidence refers to young and middle-aged adults, older people currently account for most cardiovascular disease morbidity and mortality. Our study shows that burdens of cardiovascular diseases in very elderly are even worse than in other age group or in general population^7^. Hypertension, DM, renal dysfunction, obesity, and lipid abnormalities are currently not rare in very elderly residents. Especially prevalences of some of them are relatively high. It is already reported that among very elderly American the incidence of dementia is higher than that of coronary heart disease, although both diseases are complications of cardiovascular and metabolic diseases^10^. A recent study in Shanghai community elderly and very elderly residents, which included about 600 very elderly, reported similar cardiovascular and metabolic parameter levels (SBP, DBP, FG, TG, and TC) and prevalences of hypertension and DM compared with results in our study^11^.

Based on results of this survey, hypertension is one of the most predominant health problems in very elderly Chinese in Chengdu. The proportion of very elderly with normal blood pressure (BP) is only about 6.5%. In another word, most population would suffer from hypertension when they are older than 80 years. The mean level of SBP is already as high as 146 mmHg, while levels of DBP and PP are almost the same, which is a apparent characteristic in very elderly. Also, the mean levels of glucose, UA, and creatinine are relatively high in very elderly. According to NHANES study, the prevalence of total DM increases sharply with age, from 2.4% in population aged 20–39 years to 21.6% in population aged 65 years and older^12^. It is already known that total DM prevalence in China rises from 3.2% in population aged 20–39 years to more than 21% in population aged 70 or older^13^. More than one quarter Chinese adults have two cardiovascular risk factors (i.e. smoking, overweight, hypertension, dyslipidaemia, or hyperglycaemia), and nearly one fifth adults have three risk factors^7^. In this survey, it is showed that more than 17.5% very elderly residents have ≥3 cardiovascular risk factors. Therefore, there is no doubt that the burden of cardiovascular diseases increases gradually with aging.

Compared with our previous survey in 2008, current study finds that total smoking rate in very elderly is only half of which in middle aged and elderly population (aged 40–79 years, 10.7% vs 23.7%, p < 0.001). Moreover, that difference is mainly from men (18.0% vs 54.3%, p < 0.001) while not from women (4.4% vs 4.1%, p = 0.737)^14^. Furthermore, prevalences of overweight (30.0% vs 35.1%, p = 0.001), obesity (9.4% vs 10.6%, p = 0.262) in very elderly are lower than which in middle aged and elderly population, while the prevalence of visceral obesity (54.5% vs 29.3%, p < 0.001) is much higher, which is more obviously in women. Surprisingly, the prevalence of hypertension (76.9% vs 29.4%, p < 0.001) was obviously higher in very elderly than in middle aged and elderly population. Both in very elderly men (75.6% vs 31.0%, p < 0.001) and women (78% vs 27.8%, p < 0.001), the prevalence of hypertension is two times than in middle aged and elderly population. Moreover, in very elderly, there is no obvious decrease in prevalence of DM (20.0% vs 11.3%, p < 0.001). In contrast, the prevalence of hyperuricemia increases apparently (33.0% vs 11.9%, p < 0.001). Besides, prevalences of hypercholesterolemia (35.8% vs 24.7%, p < 0.001) and hyper-low density lipoproteinemia (14.9% vs 12.0%, p = 0.009) are even higher in the elderly Chinese, while the prevalence of hypertrigleicemiea (21.6% vs 31.4%, p < 0.001) is lower. Therefore, cardiovascular risk factors distribute differently among very elderly and middle aged and elderly Chinese.

Sex differences in cardiovascular diseases are common in very elderly. Our survey demonstrates that not only prevalences of hypertension, visceral Obesity and obesity, but also prevalences of hypercholesterolaemia, hypertrigleicemiea, and hyper-low density lipoproteinemia in very elderly women are higher than in men. It seems that these abnormalities are more likely to cluster in very elderly women while not in men. Nearly one quarter of the total global disease burden is attributable to diseases in people aged 60 years and older, and half of them is in high income countries and a fifth is in low-income and middle-income countries^15^. Although China remains a developing country with the fastest acceleration in urbanization, industrialization, the burden of disease increases dramatically fast. A meta-analysis included 15 trials found that in people aged 60 years or older, 5 year event rates per 1000 people were reduced by 18 for all deaths, by 19 for cardiovascular deaths, and by 51 for combined cardiovascular morbidity and mortality by diuretics or β blockers^16^. Another meta-analysis with 14 randomized trials found that benefits from statins based cholesterol-lowering treatment were apparent for patients aged 75 years and older, with risk reduction of major vascular and coronary events^17^. Since hypertension, DM, dyslipidemia, and obesity are major modifiable risk factors for cardiovascular diseases, benefits of risk factor modification in older people are clear for control of hypertension and hypercholesterolemia.

It has been reported that UA could predict mortality in patients with heart failure^18^ and the increase of incidence of hypertension^19^. Moreover, hyperuricemia could increase the risk of cardiovascular diseases and DM in patients with hypertension^20^ and the mortality in patients with coronary heart diseases^21^. In accordance with results of previous studies above, our study finds that hyperuricemia is associated with cardiovascular diseases clustering among very elderly women.

In conclusion, prevalences of cardiovascular diseases are high among very elderly Chinese in Chengdu. Cluster of cardiovascular diseases are more apparently among vey elderly women. Hyperuricemia is associated with cardiovascular diseases clustering among very elderly women.

There are several limitations in this study. First, this is a cross-sectional study with relatively small sample, which could not describe any causality. Therefore, results of this survey should be applied with caution and longitudinal prospective cohort studies investigating the impact of hyperuricemia on cardiovascular diseases in very elderly are needed. Second, oral glucose tolerance test, hemoglobin A1c, echocardiogram, and pulse wave velocity could not been obtained in this survey because of economic reasons.

## Methods

### Participants

This cross-sectional survey was conducted from May 2013 to May 2015. Participants were recruited by using a stratified three-stage cluster sampling design. The first stage of sampling involved the random selection of five districts in Chengdu. And the second stage involved the random selection of four neighborhoods from each of the selected districts. From each of the neighborhoods, one residential committees was randomly selected.

A total number of 1056 participants from 20 residential committees were sampled according to registration data from Chengdu government. The study participants were defined as permanent residents of the households with a record in the household registration and living in local committees at least 3 years. Ethics approval was obtained from the Ethics Committee of the Second People’s Hospital of Chengdu, China. The methods in the study were in accordance with relevant guidelines and the Declaration of Helsinki. All participants gave informed consent.

### Study Design

This study was designed as a cross-sectional survey in general very elderly Chengdu residents. All participants were assessed at a survey site in their committees by well-trained researchers. In oder to avoid uncertain influences of abominable weather, the survey was not conducted when the temperature outside was below 18 °C or over 25 °C. All participants completed a questionnaire-based interview, physical examination, and electrocardiogram and fast venous blood sample taken for subsequent biochemical analysis.

The survey questionnaire included questions about basic demographic characteristics, disease history, and family history, and so on. The physical examination involved general estimation, auscultation of lung and heart, measurements of height, weight, WC, and BP (HEM-7300, Omron, Kyoto, Japan). Electrocardiogram was taken for every participant after resting for more than 15 minutes (ECG-1350P, Nihon Kohden, Japan). The biochemical analysis included FG, TC, TG, LDL-C, HDL-C, UA and creatine, which were measured in the central laboratory of the Second People’s Hospital of Chengdu.

### Abnormalities definition

Pre-hypertension was defined as 120 ≤ SBP < 140 mmHg and/or 80 ≤ DBP < 90 mmHg. Hypertension was defined as SBP ≥ 140 mmHg and/or DBP ≥ 90 mmHg and/or self-reported treatment of hypertension with antihypertensive medication in last 2 weeks according to the Chinese Guidelines on Prevention and Control of Hypertension and the Seventh Joint National Committee on Prevention, Detection, Evaluation, and Treatment of High Blood Pressure guidelines^22, 23^. Impaired fasting glucose was diagnosed if 6.1 mmol/L ≤ FG < 7.0 mmol/L. DM was diagnosed if FG ≥ 7.0 mmol/L, or FG < 7.0 mmol/L with a past history of DM^24^.

Renal dysfunction was defined as creatine ≥115 μmol/L according to the reference ranges recommend by central laboratory. Hyperuremia was defined as serum UA ≥ 416 μmol/L (7 mg/dL) in men and 357 μmol/L (6 mg/dL) in women^25, 26^. Hypercholesterolaemia was defined as TC ≥ 5.2 mmol/L according to the guidelines of Chinese adult dyslipidemia prevention and control^27^. Hypertrigleicemiea was defined as TG ≥ 1.7 mmol/L. Hyper-low density lipoproteinemia defined as LDL-C ≥ 3.4 mmol/L. Hypo-high density lipoproteinemia was defined as HDL-C level of <0.9 mmol/L in men, or HDL-C level <1.0 mmol/L in women. Dyslipidaemia was defined as any or more than one of disorders as follows: hypercholesterolaemia, hypertrigleicemiea, hyper-low density lipoproteinemia and hypo-high density lipoproteinemia. According to WHO guidelines for the Asian Pacific population, overweight was defined as 24.0 ≤ BMI <28.0 kg/m^2^, and obesity as BMI at least 28.0 kg/m^2^ ^28^. Visceral obesity was defined as WC at least 85 cm in women and at least 90 cm in men^24^.

### Statistical analysis

Data were inputted by two researchers independently and checked by a third researcher. Continuous variables were expressed as mean ± standard deviation. Frequencies were presented as percentages. Statistical comparison of continuous variables between men and women was conducted by using Student’s t -test (Mann-Whitney U test for skewed data). And x^2^ test was applied to compare frequencies between men and women. Prevalences among total elderly participants were standardized for age and sex. Stepwise multiple logistic regression models were used to evaluate the association between potential factors and disease clustering. ROC curves were used to demonstrate the sensitivity and specificity of related parameters. Calculations were performed by SPSS software (Version 17.0, SPSS Inc, Chicago, IL). A *p* value < 0.05 was considered statistically significant.
